# Managing expectations: cognitive authority and experienced control in complex healthcare processes

**DOI:** 10.1186/s12913-017-2366-1

**Published:** 2017-07-05

**Authors:** Katherine J. Hunt, Carl R. May

**Affiliations:** 1 Faculty of Health Sciences, University of Southampton, Building 67 (Nightingale), University Road, Highfield, Southampton, SO17 1BJ UK; 2 NIHR CLAHRC Wessex, Southampton, UK; 3 University Hospital Southampton NHS Foundation Trust, Southampton, UK

## Abstract

**Background:**

Balancing the normative expectations of others (accountabilities) against the personal and distributed resources available to meet them (capacity) is a ubiquitous feature of social relations in many settings. This is an important problem in the management of long–term conditions, because of widespread problems of non-adherence to treatment regimens. Using long-term conditions as an example, we set out middle range theory of this balancing work.

**Methods:**

A middle-range theory was constructed four stages. First, a qualitative elicitation study of men with heart failure was used to develop general propositions about patient and care giver experience, and about the ways that the organisation and delivery of care affected this. Second, these propositions were developed and confirmed through a systematic review of qualitative research literature. Third, theoretical propositions and constructs were built, refined and presented as a logic model associated with two main theoretical propositions. Finally, a construct validation exercise was undertaken, in which construct definitions informed reanalysis of a set of systematic reviews of studies of patient and caregiver experiences of heart failure that had been included in an earlier meta-review.

**Results:**

Cognitive Authority Theory identifies, characterises and explains negotiation processes in in which people manage their relations with the expectations of normative systems – like those encountered in the management of long-term conditions. Here, their cognitive authority is the product of an assessment of competence, trustworthiness and credibility made about a person by other participants in a healthcare process; and their experienced control is a function of the degree to which they successfully manage the external process-specific limiting factors that make it difficult to otherwise perform in their role.

**Conclusion:**

Cognitive Authority Theory assists in explaining how participants in complex social processes manage important relational aspects of inequalities in power and expertise. It can play an important part in understanding the dynamics of participation in healthcare processes. It suggests ways in which these burdens may lead to relationally induced non-adherence to treatment regimens and self-care programmes, and points to targets where intervention may reduce these adverse outcomes.

## Background

Negotiating the expectations of others is a routine part of our everyday lives. It involves us in balancing the things that they hold us accountable for (their normative expectations of us), with our ability to deliver on them (our capacity to act). Most readers of this article will have experienced such negotiations. For example, they are at the centre of the practices of appraisal and performance review that take place in universities and other corporations; they run through interactions between patients and healthcare professionals as they examine adherence to treatment regimens; and they dominate relations between the managers and owners of football teams. Our aim in this paper is to offer a middle range theory—Cognitive Authority Theory—that will facilitate understanding of the relational mechanisms involved in balancing out capacity and accountability.

As a worked example of the theory, we use the problem of balancing capacity and accountability in chronic disease management in the community. The growing epidemiological crisis over long-term (chronic) conditions, and the demographic crisis represented by an increasing proportion of older people with multiple long-term conditions [1], means that clinicians and policy makers increasingly emphasise self-care or self-management as a strategy for patient care. Here, the work of problem recognition and management, and the challenge of ongoing implementation of physical activity and changes to diet; adherence to monitoring and treatment regimens; and responding to the administrative demands of healthcare systems, are explicitly shifted to the patient and caregiver. This has led to the recognition that people experience not just the burden of symptoms, but also burden of treatment. These new burdens are a problem for people who must do the work that stems from this, often in the context of intrusive and debilitating symptoms and their psychosocial consequences [2–4]. Systematic reviews of studies of patient experience consistently reveal that this work is complex and demanding. They also reveal that the combined burdens of symptom and burdens of treatment are sometimes very hard to bear, and that they can overwhelm people with long-term conditions, with the potential for poor health outcomes as a result [5–10]. These poor outcomes may come about when patient and caregiver capacity is exceeded by the self-care workload that is delegated to them by healthcare systems and for which they are accountable [3].

The idea that adherence to treatment regimens and self-care programmes is *work* does not fit well with how we understand and evaluate engagement and participation in them. The most commonly used psychological theory in this context, is Bandura’s theory of self-efficacy. It is ‘is concerned with judgments of how well one can execute [the] courses of action required to deal with prospective situations’ [11] (p.122), and focuses on individuals’ beliefs and self-appraisals of their competence in performing specific tasks [12]. The evaluation of self-care interventions often focuses on measuring subjective self-efficacy, and associates improvements in self-efficacy with improvements in self-care. The basic proposition that underpins this kind of approach is that increased confidence in ability to perform a specific set of actions converts into sustained motivation to adhere to a structured regime of activities. This is certainly true in the short-term, and associations with a series of positive health outcomes have commonly been reported [13–16]. However, this picture is complicated because investigations tend to focus on index conditions rather than multi-morbidities, which are associated with lower levels of self-efficacy [17]. In addition, reviews of self-management programmes have detected only small to moderate increases in self-efficacy and selected health outcomes beyond 12 months [18, 19]. The reliance on self-efficacy as a primary outcome in studies of self-care and self-management programmes has meant less attention has been paid to the role of contextual and environmental mechanisms and resources in shaping patient behaviour [20]. Against this background, patient and caregiver experiences of the complexity of care need to be understood both in terms of the *work* that they do [4, 21–23], and of the ways that healthcare providers call on them to do it [24]. Interventions to improve self-care, to enhance treatment adherence, and to promote shared decision-making in the clinical encounter all depend on these encounters. Recently, researchers in this field have focused on the development of measures of treatment burden [25–29], but we also need to understand the situational and relational factors that shape it.

Our aim in this paper is to set out a theory of the behavioural and social mechanisms through which people balance capacity and accountability in relation to a set of social roles and processes. The theory responds to an important problem in the behavioural sciences: how can we best understand the dynamics of human agency under conditions of constraint [30]? This is the fundamental problem of structure and agency that runs through contemporary sociology. In this paper, we are not concerned with those elevated debates. Instead, we use self-care regimens for long-term conditions to illuminate the ways that capacity and accountability are balanced out, and show how the theory helps us to understand mechanisms that affect the sustainability of healthcare interventions for people with these conditions. It need not be restricted to these groups and these settings, however. It can be applied to any setting where inequalities of power exist. Clinical setting offer well-characterised examples of domains in which individuals negotiate their personal capacity for action whilst also interacting with institutional accountabilities and organisational expectations. Cognitive Authority Theory also contributes to understanding experiences of Burden of Treatment by identifying and characterising the factors that shape the interactional work required to adhere to self-care and other management regimens, and to access and use healthcare services. In turn, this helps us to see how patient and caregiver capacity are not fixed. Instead, they arise through negotiations about obligations.

## Methods

In this paper, we present Cognitive Authority Theory: which is a middle-range theory of the negotiation of normative expectations. It is a middle range theory because it focuses on a limited set of propositions or assumptions [31], and it is a theory and not a model or framework because these assumptions can be applied independently of context [32]. There are no uniformly accepted methods for building theory. Indeed there are diverse and conflicting ideas about what theories are [33]. In the work presented in this paper, we followed the methods successfully used previously by one of us to develop and refine theories in medical sociology, health services research and implementation science [2, 34, 35]. This comprises four sets of tasks.

**Fig. 1:**
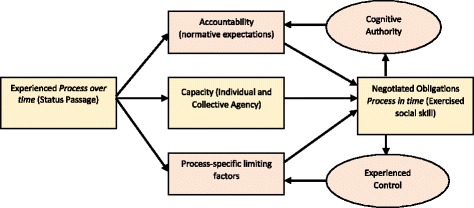
A context independent model of cognitive authority and experienced control

**Fig. 2:**
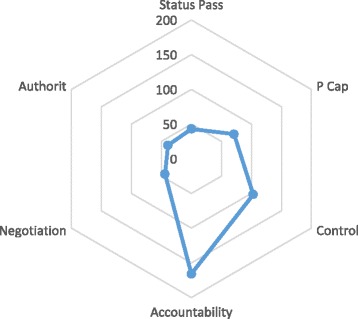
Simple counts of construct attributions derived in the construct validation phase

- ***Identification of sensitising concepts.*** First, KH undertook a small-scale elicitation study of older men’s (single phase of semi-structured interviews, *n* = 10) experiences of medical care for heart failure and comorbidities [36]. This small-scale exploratory qualitative study identified a set of behaviours related to participants’ interactions with health professionals and health care provision. This work was extended through a systematic review by Demain et al.*,* [6] that explored how patients sought to manage, and attempted to minimise, the impact of treatments on their lives in order to maintain control. This formed a set of sensitising concepts. Research Ethics Committee approval for the elicitation study described in this paper was granted by NHS (England) South Central Research Ethics Committee on 21 December 2012: REC reference: 12/SC/0638.
- ***Characterisation of theoretical constructs*** **.** Next**,** we integrated sensitising concepts derived from the elictiation study and Demain et al.’s., [6], systematic review with key elements of the growing body of theoretical and empirical literature on Normalization Processes, experiences of Cumulative Complexity, and Burden of Treatment [2, 4, 7, 29, 37–39], applied to long-term conditions.
- ***Modelling constructs*** **.** We then sifted and sorted integrated concepts, writing them as propositions that expressed generic constructs about behaviour, we linked the results of taxonomy building described above to constructs derived from already existing theories relevant to illness burden and treatment burden. This taxonomy focused attention on the *relational processes* within a healthcare system. We continued this until we reached the most parsimonious possible model of interactions between constructs. We then mapped the relationships between constructs, as shown in Fig. 1. Definitions of these constructs are provided in Table 1.
- ***Construct validation.*** The final stage of theory building was to develop a set of context-independent propositions about these relations. We then linked these together in a summary statement of the theory. This summary statement characterised and explained assumptions about the role of *cognitive authority* and *experienced control* in relational processes within a social system. We used these propositions and their related concepts as a coding framework for attribution analysis of 20 reports of patient and caregiver experiences of heart failure [40–60] that had been collected for an earlier overview of systematic reviews of qualitative studies by May et al., [10, 61]. These studies are described in Table 2., and simple counts [62] of attributions coded to the constructs of the theory are shown in Fig. 2. In Table 3, we show how sensitising concepts, theoretical constructs, and coded examples from the literature review are linked together.

**Table 1:** Definitions of key constructs of the theory

Construct	Explanation
Capacity	The affective, cognitive, informational, material, physical and relational resources that can be mobilised by individuals and groups.
Accountability	Normative expectations of actors mobilised by others.
Process Limiting Factors	External factors that challenge a person’s capacity to meet accountabilities
Negotiated obligations	A series of agreed tasks established through discussion and consensus
Experienced control	The product of an actor’s assessment of their the negotiated obligations assigned to them as practicable and the degree to which they successfully manage the external process-specific limiting factors that make it difficult to otherwise perform in their role.
Cognitive authority	The product of an assessment of competence, trustworthiness and credibility made about a person by other participants in a process.

**Table 2:** Qualitative systematic reviews experiences of Chronic Heart Failure coded in construct validation (extracted from May et al. [10, 60])

Review	Year	Type of review	Phenomena of interest
Molloy et al. [39] (UK)	2005	Mixed methods (integrative) review (16 primary studies)	Role of family caregivers in CHF
Yu [40]	2007	Qualitative systematic review (14 primary studies)	Older people’s experiences of CHF
Hopp et al. [41] (US)	2010	Integrative review (15 primary studies)	Lived experience of CHF amongst older people to inform social work practice with this group.
Barclay et al. [42] (UK)	2011	Integrative review (23 qalitative studies)	End of life care in CHF.
Dev et al. [43] (US)	2011	Qualitative metasynthesis (3 primary studies)	Self-care CHF with comorbid conditions
Dickson et al. [44] (US)	2011	Inegrative review (3 primary studies).	Self-care in CHF with comorbidities.
Kang et al. [45] (China)	2011	Qualitative metasynthesis (10 primary studies)	Role of family caregivers in CHF
Low et al. [46] (UK)	2011	Integrative review (48 primary studies)	Patient and professional understandings of disease processes and perceived needs and experiences of care provision in palliative care for CHF.
Tierney et al. [47] (UK)	2011	Qualitative systematic review (20 primary studies)	Barriers and facilitators of physical activity in CHF; beliefs and behaviors that could be targeted by interventions to promote activity.
Thomas & Clark [48] (Canada)	2011	Qualitative metasynthesis (6 primary studies)	Sex and gender related factors that shape women’s self-care beliefs and behaviors in CHF.
Clark et al. [49] (Canada)	2012	Qualitative metasynthesis (58 primary studies)	Factors and processes associated with help-seeking decisions in CHF.
Jani et al. [50] (UK)	2012	Qualitative systematic review with framework analysis (16 primary studies)	Treatment burden in CHF at end of life.
Procter [51] (UK)	2012	Qualitative systematic review (5 primary studies)	Contribution of palliative care specialists to end of life care in CHF; barriers to collaborative clinician-patient relations; and patient and carer expectations and needs.
Buck et al. [52] (Canada)	2013	Integrative review (30 primary studies)	Specific activities by which caregivers contribute to self-care beliefs and behaviors in CHF
Falk et al. [53] (Sweden)	2013	Mixed methods (Integrative) review (23 primary studies)	Lived experience of self-reported symptoms, illness experience, and self-care management by older patients with CHF
Siabani et al. [54] Australia)	2013	Qualitative metasynthesis (23 primary studies)	Factors that prevent optimal engagement with self-care regimens in CHF
Sookhoo et al. [55] (UK)	2013	Qualitative metasynthesis (8 primary studies)	Participation in CHF self-management education programs for CHF
Clark et al. [56] (Canada)	2014	Qualitative metasynthesis (49 primary studies)	Patients and caregivers’ perceptions of effective self-care in CHF
Dekker [57] (US)	2014	Qualitative systematic review (13 primary studie)	Experiences of depressive symptoms in CHF
Harkness et al. [52] (Canada)	2014	Qualitative metasynthesis (47 primary studies)	Strategies for self-care in everyday life
Strachan et al. [58] (Canada)	2014	Qualitative metasynthesis (45 primary studies)	Contextual factors that influence self-care in CHF
Wingham et al. (UK) [59]	2014	Meta-ethnography (19 primary studies)	Attitudes, beliefs, expectations and experiences of self-management in CHF

**Table 3:** Sensitising concepts, theory constructs and examples from the literature

Sensitising Concepts	Initial data sources ES = Elicitation study; SR = Demain et al. [6] Systematic Review	Theory Constructs	Examples extracted from Heart Failure Studies included in May et al. [10, 60] systematic review – Construct Validation
Status passage	SR	Status passage	‘Several studies described adjustment to living with CHF as a process. Stull et al. described the entire process of living with CHF as a process of searching for meaning and identity, which started from a ‘crisis event’, followed by phases of ‘diagnosis’, ‘patient’s responses to the diagnosis’, ‘acceptance and adjustment’ and ‘getting on with life’. The ‘crisis event’ described the patient’s perception of the initial manifestation of CHF that placed them in a new and uncertain situation. In the phase of ‘diagnosis’, patients with CHF tried to make sense of their situations by attaching meanings to the symptoms. In this phase, patients relied on prior experiences with similar situations to make sense of the cues in their current situation. The process of searching for new meaning was, however, greatly hindered by fluctuations in their debilitating symptoms, the concomitant hospital admissions, the disruption to their usual role in life and identity, and the limited treatment options’ [40].
Available agency	ES	Capacity	‘Action-based strategies also included enlisting the help of caregivers for assistance with self-care activities. Caregiver assistance ranged from simple reminding to taking over some of the responsibilities such as organizing medications, buying groceries and preparing meals according to dietary guidelines, monitoring symptoms, and navigating the healthcare system as needed. Although some patients felt they did not want to be a burden to caregivers, at the same time they recognized their inability to manage self-care activities without caregiver help’ [104].
Help-seeking	ES, SR		
Contribution	ES		
Informal/Unwritten contracts	SR, ES	Accountability	‘Individuals who were able to assimilate formal knowledge accurately and adapt their lives accordingly, while recognising the uncertainty of HF. Advanced self-managers tend to be better educated than those who adopted the above approaches. A distinguishing feature of advanced managers was their understanding of and willingness to be constantly vigilant about their physical and mental state and desire to be in control of their management. They were also able to adapt their medication as they perceived necessary and were keen to manage their own symptoms or to improve participation in social activities. Advanced self-managers also recognised the importance of family members and were mindful that decisions of care also impacted on them’. [59].
Hierarchical relations	SR		
Treatment workload	SR		
Judgments about the competence of self	ES		
Medical dominance	ES, SR	Process limiting factors	‘A number of other barriers were identified as factors hindering adherence, including patient knowledge deficits, physical limitations, financial hardship, low motivation or negative experiences or beliefs toward treatment, limited self-efficacy, and difficulty coping. Follow-up attendance was limited by patients’ difficulty getting to the hospital, including the cost of transportation, problems with public transit, intolerance of crowds, and the inability to walk long required distances’ [78]. ‘Financial challenges were consistently reported by patients to be a barrier to self-care, particularly in relation to diet and medication management. High costs of medications and healthy foods competed with other life demands, at times straining patients’ ability to meet even their basic needs. Patients could engage in a trade-off where the needs and costs of daily life were prioritized over adherence to self-care, requiring them to make difficult financial and health decisions without health professional advice. For example, patients chose to fill certain prescriptions over others or to skip or reduce their doses of medications. This was particularly true for patients who required insurance to access treatment, as this quote so eloquently captures: “The doctor gives you 6 prescriptions and Medicaid only pays for 3, so what happens with the other 3? In that case I just don’t buy them” [58].
Gatekeeping and rationing	ES, SR		
Disruption	ES		
Burnout	ES		
Structurally induced non-adherence	SR		
Diffusion of responsibility	ES	Negotiated Obligations	‘Studies also reported poor and inappropriate care practice, in terms of health professionals not engaging patients in their care and decision making, patients not receiving sufficient information about diagnosis or condition management, insensitive approaches to female patient needs, and improper medication scheduling. Other examples of poor care practice included health providers creating unnecessary fears, not tending to immediate needs such as toileting assistance and ignoring patients. When patients experienced poor quality of care they reported lack of confidence in care providers, confusion, delays in seeking care and were deterred from maintaining positive self-care practices. Naturally, prior negative experience of accessing services discouraged patients from seeking timely help’ [67]. ‘Although effective communication with health professionals was seen to be a key to high quality care, patients perceived that health professionals exercised ‘information holding’. They also reported health professionals: did not listen, provided inconsistent or vague information, did not follow up and had poor communication with other health professionals. The need for better explanations of heart failure and its symptoms from professionals were widely noted. Some health professionals recognized the need for good communication with patients, but were constrained by lack of time in consultations. Patients could perceive that professionals had exclusive responsibility for management/symptom monitoring or that responsibility was shared’ [49].
Opportunity structures			
Agreeing expectations	ES, SR		
Agreements about collaboration	ES, SR		
Social Skill	SR		
Competency assessment	ES	Cognitive authority	‘Patients often feel disempowered, finding clinicians unapproachable and reluctant to give information: they may see questions about prognosis as taboo be reluctant to ask questions, especially if older, be unsure what questions to ask, be afraid to ‘put the doctor on the spot’, and fear being seen as difficult, demanding, or complaining. Some hesitate to visit a doctor, fearing unwelcome and unwanted hospital admission, or find themselves too fatigued and unwell to be able to concentrate and absorb information’ [42].
Self-surveillance	ES		
Patients reject medical authority	ES		
Calculation of options	ES, SR		
Judgments about the competence of others	ES, SR		
Patients call for specialist not generalist help	ES	Experienced control	‘Patients losing a sense of control over their illness were reported as connecting the loss of control with unpredictable deterioration in health, high blood pressure, shortness of breath and sleeplessness and over their life in terms of loss of independence, financial security and participation in CHF management decision-making . Losing this sense of control, or ‘feeling imprisoned in illness’, as Ekman described, was also associated with various restrictions imposed on their lives due to the need to adhere to disease management, resulting in feelings of helplessness, powerlessness and that premature death was unavoidable’ [67].
Rational non-adherence	SR		
Adaptive work	ES, SR		

## Results: Cognitive authority and experienced control

Cognitive Authority Theory assists in understanding the ways that people negotiate complex relational processes in conditions marked by unequal social relations. We developed the theory through two assumptions that suggest how (less powerful) patients and caregivers interact with (more powerful) healthcare providers and organisations. First, we are concerned with the *cognitive* authority of actors in a complex social process. Cognitive authority itself is the product of an assessment of competence, trustworthiness and credibility made about a person by other participants in a process.

(i) *The degree of cognitive authority possessed by an actor in a complex social process depends on balancing their available capacity against the expectations of others.*

Second, we are concerned with the extent to which a person assesses their obligations within their role as ‘doable’ in the context of their life-world and experience; and the degree to which they successfully manage the external process-specific limiting factors that make it difficult to otherwise perform in their role. That is, the extent of their experienced control.

(ii) *Experienced control over a process depends on participants’ ability to negotiate and manage process-specific limiting factors that challenge their capacity to achieve goals.*

In what follows, we unpack these two assumptions: defining accountabilities, capacity, and process-specific limiting factors, and then developing the concepts of cognitive authority and experienced control. Because our theory is context-independent, we distinguish between two general classes of participants in these relational processes. These are *institutional actors* (who can be considered more powerful by virtue of the authority derived from their institutional position, and who include healthcare providers), and *population actors* (who can be considered less powerful because they are expected to defer to institutional actors, and include patients and caregivers).

To connect these constructs with real-world processes, we focus on aspects of patient and caregiver experiences of long-term life-limiting conditions. We draw on recent studies of experiences of such conditions through revisiting a meta-review of systematic reviews of qualitative studies of patient experiences of heart failure, chronic obstructive pulmonary disease, and chronic kidney failure [10, 61]. Interactions between patients, caregivers, healthcare professionals, policy-makers, and services are an excellent vehicle for understanding participation in complex relational processes.

### Patient goals and illness careers

The purpose of Cognitive Authority Theory is to help us make sense of the relationships between actors, as they negotiate a process, within a dynamic social system. By social system, we mean that they are in an organised pattern of social relations in which participants have broad awareness of the rules and resources that inform behaviour within it, have broad awareness of the goals of others within it, and share relative agreement about the appropriate ways of achieving those goals. Taking this approach enables us to deal with the problem of agency under structural constraints, noted at the beginning of the paper. Fligstein has drawn on interactionist perspectives to characterise these behaviours as ‘social skill’ [63].

Social skill can be defined as the ability to induce cooperation among others. Skilled social actors empathetically relate to the situations of other people and, in doing so, are able to provide those people with reasons to cooperate (…). Skilled social actors must understand how the sets of actors in their group view their multiple conceptions of interest and identity and how those in external groups do as well. They use this understanding in particular situations to provide an interpretation of the situation and frame courses of action that appeal to existing interests and identities (p.112).

Within such systems, we are interested in social processes characterised by negotiations about compliance and obligations within a set of normative expectations of action, experienced under conditions of constraint. In these negotiations, participants relate these expectations to (i) individual and distributed capacity to participate, and (ii) the contexts in which such action takes place and which present participants with process-specific limiting factors. When they do so, it is often within the context of a temporal process defined by *status passage* [64].

A status passage occurs when individuals, or groups, experience institutionally and organisationally ascribed transitions over time. In these transitions, identities are redefined, and interactions and relations with others are reconfigured. Following from this come changed normative expectations about their roles and actions and, in turn, changes in the ways that others respond to them. Status passages exemplify *a change in state over time*—the most fundamental definition of a process—and they rely on shared definitions of the legitimacy of role changes, and shared understandings of the nature of the underlying reconfiguration of identity.

In long-term conditions, reviews demonstrate consistent evidence to the effects of such changes in experienced identity: these may include reduced self-esteem and self-worth, and loss of social functioning [49, 58, 65–69]. The effects of these changes can be powerful, and they include fear, anxiety, isolation and discomfort [48, 50, 55, 58, 70, 71]. Awareness of the meaning of disease and it implications frame the ways that people interact with services [43, 47, 67, 72, 73]. At the same time, those anxieties could be ameliorated by finding ways to improve the ways that patients and caregivers could influence the course and direction of their passage through care [71, 72, 74, 75].

### Unequal negotiations between population and institutional actors

We have already observed that participants in relational processes can be treated as though they are members of two groups. Here, more powerful actors possess institutional or organisational qualities that empower them to impose accountabilities and normative expectations of others. These expectations have institutional or organisational sources; they are generalised across groups or populations though implicit theories that characterise the identities and explain the behaviours of less powerful actors. Against this background, the possibilities for the exercise of social skill (i.e. negotiating towards goals), by population actors depends on the degree of cognitive authority that they can negotiate with institutional ones. We define cognitive authority as the extent to which population actors are seen by others to possess qualities of competence, trustworthiness and credibility in meeting the accountabilities and accomplishing the tasks implicated in a relational process. Cognitive authority forms an important resource for actors when these are involved in negotiations about their participation in an institutionally defined process. It rests on the extent to which more powerful actors accept the experiential claims that less powerful actors make about their state and how this affects their available *capacity* to participate in that process and thus meet their accountabilities*.* By capacity, we mean the extent to which less powerful actors can access and mobilize individual and group resources. These resources may have affective, cognitive, informational, material, physical and relational components.

Studies of the lived experience of chronic or long-term conditions consistently show how interactions between these two classes of actors are formed and worked out in real-world settings. Patients and caregivers live with disease processes that are governed by biological mechanisms of pathophysiological deterioration, and in which they experience and make sense of symptoms and effects of disease, and the effects of treatments [76]. These processes are given meaning by social mechanisms of status passage, in which they experience and make sense of changes in social identity and status that are attributed to them by others [64]. Health system responses to these diseases aim to retard, or at least stabilise, disease progression. They promote health-related behaviour change such as diet and exercise [77]; weight loss [56]; smoking cessation [78]. They also enrol patients and caregivers into a set of delegated activities (symptom recognition and monitoring [79]; medication adherence and management [51]; participation in rehabilitation programmes [70]; and the operation of health technologies [80]. The normative assumption that underpins these interventions is that the patient – and caregiver – will be motivated to engage in behaviour change and to participate in delegated clinical activities.

Internationally, there is now a mass of policy that stresses that motivated and adaptive participation in self-care is a key element of control over disease progression [81]. Patient and caregiver capacity is the necessary foundation for this [82]. While empirical studies do much to reveal the ways in which normative expectations of participation are played out, they also show how patient and caregiver capacity is formed and expended. Indeed, caregivers’ solidarity seems to be central to this process [40, 53, 65, 83]. It supports symptom recognition and effective engagement with services [53, 55, 84, 85], but diminishes when caregiver workload interferes with normal life [40, 41, 74, 75, 80, 86].

### Process limiting factors

Cognitive authority is the product of negotiations about *agency—*available capacity and its exercise—between population and institutional actors. However, these relations are often situated in contexts where neither party may be able to control the course or direction of a process, and where capacity and accountability are constrained by the intervention of external mechanisms that affect them. External mechanisms take the form of *process-specific limiting factors* when they intervene in ways that change participants’ actions within a relational process. Actors must develop and negotiate coping strategies to successfully overcome those external factors and *experience control* over the effects of what may be the result of interactions between irreversible pathophysiological processes (e.g. disease progression and its effects), and large-scale structural mechanisms (e.g. resource allocation and organisation in healthcare systems). These define the rate of change in a relational process, and direct the course of important events within it. Here, patient and caregiver *capacity* is equally diminished by their access to services and other resources [70, 79, 86, 87], and by poor professional support, co-ordination and responsiveness, and continuity of care [44, 51, 68, 70, 79, 86]. Indeed, patients and formal and informal caregivers are often disadvantaged by poorly communicated information about disease processes and symptoms [45, 55, 67, 72, 88, 89]. Although pathophysiological and cognitive deterioration matter a great deal [90], it is often clinician behaviours that matter more. For example, these may include reluctance to discuss the life-limiting nature of heart failure and to inform patients and caregivers about the meaning of symptoms [43].

Relations between population and institutional actors that underpin negotiations that inform experienced control over a relational process are rarely equal. One is empowered to define the ways in which the other exercises its capacity, and to hold it accountable for what it does. Unstable and unequal relations are fertile ground for complex interactions between system regulators and system subjects, and they are framed by a set of power relations through which rules that govern behaviour [91], and the roles and relative positions of participants [92], are defined and enacted. In the relations on which this paper focuses—those between population and institutional actors participating in self-care regimes—there is a substantial body of theory already that characterises the relative powers of patients and clinicians through medical dominance theory [93, 94]; identity theory [95]; and status passage theory [96]. Patients and caregivers cannot negotiate with healthcare professionals who are reluctant to communicate openly about their condition [43, 97, 98], or who impute accountabilities to them that they have no capacity to meet [99].

## Discussion

**Fig. 3:**
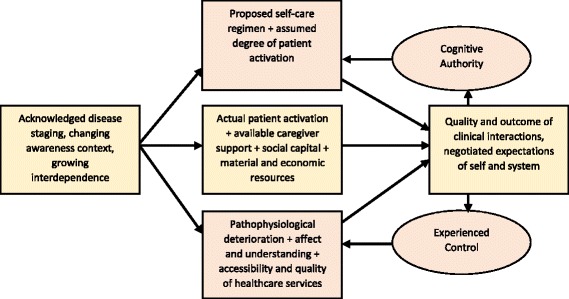
Cognitive authority: complex interactions between experienced status passage and consequences of clinical encounters

We have outlined Cognitive Authority Theory: a middle-range theory that describes and explains mechanisms through which actors seek to exercise social skill within a status passage. Here cognitive authority and experienced control moderate population actors’ experiences of both their accountabilities (the normative expectations of institutional actors), and process-specific limiting factors (other relational and structural mechanisms that intrude into and externally shape status passage). We mapped the constructs of the theory in Fig. 1. To illustrate how the theory works, we have drawn on the wider literature that examines patient experiences of life-limiting long-term conditions, and on qualitative data collected in a small-scale elicitation study. In Fig. 3, we mapped the constructs of the theory directly on to those experiences. In this form, our theory helps to bridge the gap between three higher-level theories: Bandura’s social psychological theory of agency [12] and self-efficacy [11]; Fligstein’s theory of social skill as inducing co-operation in dynamic social systems [63]; and Glaser and Strauss’ theory of status passages as dynamic processes of identity formation and negotiation [64].

### Summary statement of the theory

In summary, Cognitive Authority Theory rests on the following assumptions.

- The degree to which population actors implicated in a complex social process possess Cognitive Authority depends on them balancing their available capacity against the expectations of others. Their experienced control over a situation or process depends on their ability to negotiate and manage process-specific limiting factors that challenge their capacity to achieve goals.
- Cognitive Authority and Experienced Control are mediated by actors’ *capacity*, defined as the extent to which an actor possesses and can mobilize personal resources, (which may be affective, cognitive, informational, material, physical and relational), in the service of those goals; and by their *accountability*, defined as the extent to which others impute to the actor the ability to mobilize those resources to meet their expectations.
- Capacity and accountability are moderated by interaction processes that lead to *negotiated obligations*. These negotiated obligations may be temporary, but they provide a normative structure for future action because they define the degree of accountability that can be imputed to an actor.

These assumptions lead the theory to propose that:

(d) When population actors in a complex relational process are faced with normative expectations of institutional actors that exceed their perceived capacity, they will seek opportunities to negotiate the *relational* balance between capacity and accountability.
(e) In relation to *(d)*, the theory proposes that the cognitive authority possessed by a population actor depends on their degree of success in balancing their available capacity against the accountabilities imputed to them by institutional actors; and that the degree of sustained motivation to meet negotiated accountabilities is proportionate to the cognitive authority possessed by population actors.
(f) When population actors in a complex relational process are faced with demands of process-specific limiting factors that exceed their perceived capacity, they will seek opportunities to negotiate the *practical* balance between capacity and practical demands.
(g) In relation to *(f)*, the theory proposes that the degree of experienced control possessed by a population actor depends on their degree of success in balancing their available capacity against the constraining effects of the contexts in which these tasks must be undertaken; and that the degree of sustained motivation to meet negotiated accountabilities is proportionate to population actors’ experienced control over process-specific limiting factors.

### The problem of relationally induced non-adherence

Given the empirical focus of our account of Cognitive Authority Theory, it is not surprising that one important area for its application is in understanding the management of long-term or chronic conditions. As we observed earlier in the paper, research framed by Normalization Process Theory [39], the Cumulative Complexity Model [4], and Burden of Treatment Theory [2], has focused on understanding the relationship between the workload that healthcare providers ask patients to take on, and the patient’s capacity to do this work. This has led to the notion that the diminution of patient participation in self-care programmes and adherence to treatment regimens over time may be *structurally induced* [3], when patients and caregivers become overburdened by the demands of care. Put simply, workload becomes too much to manage. Cognitive Authority Theory suggests that there is another dimension to patient experience that is not fully accounted for in either the Cumulative Complexity Model or Burden of Treatment Theory. This is that the mechanisms that lead to diminution in participation in self-care programmes and treatment regimens over time have an important *relational* component.

The concept of relationally induced non-adherence is potentially important. Like structurally induced non-adherence, it is iatrogenic. It may arise when institutional actors either cannot or will not enter into negotiations over population actors’ beliefs or knowledge about what is possible within the context of an experienced process. In healthcare, this means that the patient’s claim to cognitive authority is denied, (and it is not far from this to a deficit model of patient beliefs and behaviours), and their attempts to exercise experienced control over process-limiting factors may be complicated by paternalistic models of care.

Patients, caregivers and health professionals vary in their need and desire for authority and control [100] and research has shown that agreement on healthcare responsibilities between patients and health professionals is associated with better health outcomes [101]. We propose that the negotiation of *doable* tasks and reasonable obligations positively influences cognitive authority and experienced control. Clinical tools already exist that can be applied to the problem of relationally induced non-adherence to treatment regimens and programme participation. These include high quality shared decision-making tools [102, 103], and consultation models of deliberative and collaborative encounters between patients, caregivers, and healthcare providers [104].

## Conclusion

The theory of Cognitive Authority presented in this paper helps us understand key mechanisms involved in configuring people’s experiences of long-term life limiting conditions. Although we have applied it to experiences of long-term conditions, it is context-independent in its fundamentals, and can be applied to unequally formed relations between population (less powerful) and institutional (more powerful) actors in a wide variety of situations. In this paper, we have shown how the theory can be used to explore the relational content of patient *work* in the management of long-term conditions. This has important implications for healthcare provider organisations, for whom structurally or relationally induced non-adherence is a potential problem. It highlights the importance of implementing shared decision-making techniques in the clinical encounter in ways that enable clinicians, patients and caregivers to focus on achievable and sustainable objectives. Beyond this, it provides a way of showing the effects of normative expectations expressed by healthcare providers and policy-makers. When these fit poorly with patients’ and caregivers’ actual capacity for participation, and when they fail to take into account the effects of system level limiting mechanisms, then non-adherence to treatment regimens can be *relationally induced*. Better understanding of the processes and mechanisms characterised in this paper will lead to ways to improve support for patients and healthcare professionals in making help-seeking behaviours more appropriately focused, and self-care regimens more sustainable.

We have focused on the ways that Cognitive Authority Theory explains important aspects on relations between people with long-term conditions and clinicians. There is no reason, however, to restrict it to this. At the beginning of this paper, we made the point that negotiating normative expectations is ubiquitous in social relations. The approach we have presented here can be readily applied to relations between health professionals, between patients and caregivers—or between participants in any set of organised social relations where inequalities of power are negotiated. Cognitive Authority Theory explains (a) mechanisms that shape experienced control over process-specific limiting factors, within a process characterised by status passage, and (b) the role of the cognitive authority of participants in motivating and shaping that process. Future empirical research will refine its constructs and test its predictions
